# The impact of nanodrugs on the metagenome of tobacco rhizosphere soil

**DOI:** 10.3389/fmicb.2025.1715400

**Published:** 2026-02-11

**Authors:** Chun-Mei Lai, Xiao-Shan Xiao, Li-Wei Liu, Xue-Li Li, Yu-Wei Luo, Yan-Qi Liang, Yan Cheng, Yuan Qin

**Affiliations:** Fujian Provincial Key Laboratory of Haixia Applied Plant Systems Biology, College of Life Sciences, College of Plant Protection, Fujian Agriculture and Forestry University, Fuzhou, China

**Keywords:** metagenomics, micro-ecosystem, nanodrugs, rhizosphere microorganisms, tobacco

## Abstract

The occurrence of tobacco diseases seriously restricts the healthy development of the tobacco industry. Soil microorganisms play an important role in regulating ecosystem functions. However, the impact of nanodrugs on the rhizosphere microbial community of tobacco and its related functions is still unclear. Therefore, this study combined field experiments to evaluate the effect of nanodrugs in reducing diseases and combined metagenomic sequencing to further explore the micro-ecological mechanism of nanodrugs in stably reducing soil biological barriers. The results show that nanodrugs can significantly improve the health level of tobacco. Metagenomic sequencing found that nanodrugs treatment increased the diversity and abundance of bacterial communities and could regulate the structure of soil microbial communities. It could selectively recruit beneficial microorganisms such as *Sphingomonas*, *Bradyrhizobium*, *Pseudomonas*, and *Nocardioides* to assist tobacco in disease control. GO function analysis showed that nanodrug treatment groups had significant enrichment of energy metabolism-related functions such as electron transfer activity, ATPase activity, and redox processes. KEGG pathway analysis showed that the relative abundance of key metabolic pathways such as fatty acid metabolism, aminoacyl-tRNA biosynthesis, ribosome, and purine metabolism was significantly increased. This study found that nanodrugs may indirectly promote plant health and alleviate tobacco diseases by shaping microbial community structure, enriching beneficial bacterial communities, and activating key metabolic pathways. These findings provide a theoretical basis for the application of NMs in the regulation of agricultural micro-ecosystems.

## 1 Introduction

Tobacco (Latin name: *Nicotiana tabacum* L.), also known as tobacco leaf, is an annual herbaceous plant reaching up to 2 meters in height. Leaves are oblanceolate, lanceolate, oblong, or ovate; petioles are inconspicuous or winged. Capsules are ovoid or ellipsoid; seeds are orbicular or broadly oblong-rotund, brownish. Flowering and fruiting occur from summer to autumn. This species can be utilized as a raw material in the tobacco industry, which has emerged as one of the world’s most economically vital crops due to its global significance (Chen et al., 2023). Yunnan Province, accounting for approximately 50% of China’s total tobacco output, stands as the nation’s major tobacco-producing region (Li et al., 2019). As a critical economic crop, the tobacco industry generates substantial fiscal revenue for the national treasury through taxation, underpinning China’s stable and rapid socio-economic development. However, in recent years, persistent outbreaks of diseases such as tobacco mosaic virus (TMV), black shank, and hollow stalk disease have not only caused a sharp decline in tobacco yield but also severely impaired leaf quality, inflicting significant economic losses on the sector.

Currently, conventional plant disease control methods are diverse, encompassing breeding disease-resistant varieties, implementing rational crop rotation, applying biological control, and using chemical interventions. Among these, crop rotation is a common agricultural practice that can modulate the dynamics of metabolites in the tobacco rhizosphere, thereby impacting soil fertility (Zhou et al., 2023). The network structure of soil microbiomes is closely intertwined with nutrient cycling and crop growth in agricultural ecosystems. Factors such as available potassium, available phosphorus, pH, and sampling period in rhizosphere soil significantly shape rhizosphere microbial communities. For instance, ryegrass tillage and incorporation can alter soil microbial assemblages-increasing the abundance of pathogen-antagonistic microorganisms (such as *Lysobacter*, *Sphingomonas*, *Chaetomium*, and *Minimidochium*) while reducing the prevalence of the pathogenic fungal genus *Neoconiospora* (Zhou et al., 2023). With advancements in agricultural technology, there is growing interest in exploring more efficient and environmentally friendly disease management strategies. Among these, nanomedicines have emerged as a promising alternative to traditional agents in tobacco disease control, owing to their high efficacy, low toxicity, strong targeting ability, and environmental compatibility-offering novel solutions for modern agriculture. Nanomaterials (NMs) are defined as materials with at least one dimension in the 1–100 nm nanoscale range (Miernicki et al., 2019). When introduced into soil, NMs interact directly and indirectly with soil microbial communities-a group of organisms critical to regulating and maintaining multiple soil functions (Hartmann and Six, 2023). Nanomedicines not only control plant diseases but also improve soil microbial ecology, enhance pesticide use efficiency, strengthen disease suppression efficacy, reduce chemical inputs, minimize environmental impacts and residue risks, and exhibit sustained-release long-acting properties. Additionally, they decrease application frequency, boost tobacco’s inherent disease resistance, and achieve a synergistic combination of prevention and treatment. Furthermore, NMs display high adaptability, enabling them to address complex disease scenarios. Major tobacco diseases include tobacco mosaic virus (TMV), bacterial wilt (*Ralstonia solanacearum*), and black shank (*Phytophthora nicotianae*). Existing studies have revealed distinct differences in rhizosphere soil/root characteristics and microbial composition between healthy tobacco and *Ralstonia solanacearum*-infected plants, with higher relative abundances of pathogenic bacteria in diseased individuals (Muzammil et al., 2023; Andreadelli et al., 2021; Wang et al., 2022). Currently, global research on nanomedicines in agriculture focuses primarily on inhibiting pathogen growth, promoting crop development, controlling plant diseases, and alleviating abiotic stress (Ali et al., 2024; Cai et al., 2018; Faiz et al., 2022).

Based on this rationale, we herein developed a self-assembly nano delivery system (QCu^2+^ NPs) fabricated by using the quaternary ammonium chitosan (QAC) and copper ions (Cu^2+^) as raw materials. Subsequently, the resultant was applied to tobacco rhizosphere soils at three gradient concentrations (100, 150 and 200 μg/mL^−1^) to simultaneously evaluate its efficacy against *R. solanacearum* induced bacterial wilt and its potential side effects on the below-ground micro-ecosystem. Employing high-throughput 16S rRNA amplicon sequencing, we systematically dissected shifts in microbial community structure and network topology under each dosage, aiming to elucidate the trade-off between antibacterial performance and ecological safety. We hypothesize that QCu^2+^ NPs first modulate soil physiochemical properties, thereby driving the re-assembly of microbial communities; keystone taxa and their associated network attributes subsequently govern soil functional outputs, ultimately generating predictive power for tobacco yield (Qiao et al., 2024). Integrating rhizosphere metabolomic profiles with microbial functional information will further unravel the coupling mechanisms among microbial diversity, metabolite landscapes and ecosystem functioning (Holden, 2019), providing a theoretical framework for establishing a nano-enabled, green tobacco production system that couple disease management with soil-health improvement and sustainable agricultural development.

## 2 Materials and methods

### 2.1 Synthesis of QCu^2+^ NPs

This experiment employed the nanoprecipitation method. First, 0.1 g of QAC powder was accurately weighed and placed in a 15 mL centrifuge tube, followed by the addition of 10 mL of ultrapure water solution. The mixture was then incubated in a horizontal vertical rotary mixer for 30–60 min until complete dissolution, yielding a clarified QAC solution. A clear 10 mg/mL CuCl₂ solution was prepared using the same method. Subsequently, 200 μL of the 10 mg/mL QAC solution was taken and added dropwise to 700 μL of ddH₂O under vortexing, followed by vortexing for 15–30 s to obtain QAC NPs. Next, 100 μL of the 10 mg/mL CuCl₂ solution was gradually added dropwise to the QAC NPs and vortexed for the same duration (15–30 s), resulting in 100 μg/mL QCu^2+^ NPs. Similarly, QCu^2+^ NPs at concentrations of 150 μg/mL and 200 μg/mL were prepared for subsequent field experiments.

### 2.2 Experimental materials and design

The experiment was conducted in 2025 in the tobacco field of Qingxi Village, Taixi Township, Youxi County, Sanming City, Fujian Province (118.26208°E, 26.05713°N). Tobacco plants with uniform growth were selected as the test materials. The study investigated the effects of different nanodrug concentrations on the rhizosphere microbial community of tobacco plants using the same soil for root irrigation. The experiment included four treatments: CK group (water treatment), Experimental Group A (100 μg/mL nanodrug treatment), Experimental Group B (150 μg/mL nanodrug treatment), and Experimental Group C (200 μg/mL nanodrug treatment). Each treatment had three replicates, and each replicate consisted of 30 tobacco plants. The roots were irrigated with different concentrations of nanodrug solutions, with irrigation conducted every 5 days for a total of 30 days. During the experiment, all other management measures were strictly maintained the same across all treatments.

### 2.3 Soil sample collection

Soil was collected from the lower layer of the tobacco roots, specifically from a depth of 0–20 cm. The soil samples were then carefully sieved through a 2 mm mesh to remove any stones and root fragments. The sieved soil samples were subsequently stored at −80 °C for subsequent metagenomic sequencing.

### 2.4 Metagenomic sequencing analysis of soil

Microbial DNA was isolated and extracted from soil samples using a specific kit. The DNA was fragmented using a mechanical disruption method (ultrasonication). Subsequently, the fragmented DNA was purified, end-repaired, and an “A” base was added to the 3′ end. Sequencing adapters were then ligated to the DNA fragments. Agarose gel electrophoresis was used to select fragments of the desired size. PCR amplification was performed to generate paired-end libraries. After library quality control, high-throughput sequencing was conducted using the NovaSeq 6,000 platform with a PE150 sequencing mode.

### 2.5 Metagenomic sequencing analysis of soil

The raw data was preprocessed using fastp to obtain high-quality data for subsequent analyses. Further host sequence filtering was conducted to acquire valid data (ValidData). The valid data for each sample was assembled using the MEGAHIT software. Based on the assembly results, the QUAST program was employed to evaluate the assembly outcomes (Mikheenko et al., 2023). The MetaGeneMark software was utilized to predict CDS (Coding Regions) for the assembled contigs (≥500 bp) from each sample, and sequences with CDS lengths less than 100 nt were filtered out based on the prediction results. Subsequently, the MMseqs2 software was used to remove redundancy from the CDS predictions, yielding a set of non-redundant Unigenes. The DIAMOND software was then employed to compare the Unigenes protein sequences with the NR meta database to extract a sub-library of NR meta for species annotation (Duan et al., 2020). Species diversity was analyzed using methods such as Alpha diversity analysis, PCoA analysis, NMDS analysis, UPGMA analysis, and Anosim analysis. Functional annotations of the samples were carried out using general databases like GO, KEGG, eggNOG, MCycDB, PCyCDB, SCycDB, NCycDB, AsgeneDB, and FeGenie (Quince et al., 2017), as well as specialized databases like CARD and CAZy for functional annotations.

### 2.6 Data processing

Data statistics and analysis were conducted using Excel 2022 and SPSS 24.0. One-way ANOVA tests were employed for analysis, and LSD (Least Significant Difference) method was used for multiple comparisons of the data. Additionally, sequencing experiment data analysis was performed on the OmicStudio Cloud Platform (https://www.omicstudio.cn/), and Origin Pro 2024 was used for data visualization and graphing.

## 3 Results and discussion

For the individual components of the nanomaterial, the hydrodynamic diameter of pure CuCl₂ was 142 nm, while the average hydrodynamic diameter of QAC was 275 nm. The final QCu^2+^ NPs exhibited an average hydrodynamic diameter of 237.5 nm (Supplementary Figure S1).

### 3.1 Gene prediction

We used the MetaGeneMark software to predict CDS (Coding Regions) for the assembled contigs (≥500 bp) from each sample and filtered out sequences with CDS lengths less than 100 nt based on the prediction results. Subsequently, based on the CDS prediction results, we used the MMseqs2 software to remove redundancy and obtain a set of non-redundant Unigenes. Finally, we used Bowtie2 to align the CleanData (or ValidData after host sequence removal) of each sample to the Unigene sequences, calculated the number of reads aligned to each Unigene in each sample, and filtered out Unigenes with ≤2 aligned reads in all samples, obtaining the final Unigenes for subsequent analysis.

Based on the number of aligned reads and the each of sample Unigene, the number of each Unigene in each sample was calculated using the following formula:


$$G_{k}={\frac{r_{k}}{L_{k}}}^{*}{\frac{1}{{\sum^{n}}_{i=1}\frac{r_{k}}{L_{k}}}}^{*}10^{2}$$

Where r represents the number of reads aligned to the Unigene, and L represents the length of the Unigene.

As indicated by the Unigene length analysis values in Table 1, significant differences in the number of Unigenes in each sample were observed after the rhizosphere soil of tobacco was treated with different concentrations of nanodrugs (treatments A, B, and C). Specifically, the number of Unigenes in the low-concentration (A) nanodrug treatment group was significantly better than that in the medium-concentration and high-concentration nanodrug treatment groups, suggesting that low-concentration nanodrugs can enhance the transcriptional activity of soil microorganisms, whereas high concentrations tend to suppress their diversity. The primary reasons for these observations are multifaceted. First, low concentrations of nanodrugs may provide a more favorable environment for microbial growth, acting as a mild stimulant that promotes the activity of beneficial microorganisms in the soil. Conversely, higher concentrations of nanodrugs may introduce toxicity to the soil environment, disrupting the normal physiological functions of microorganisms and leading to stress responses and reduced growth rates. Additionally, different concentrations of nanodrugs exert varying levels of selective pressure on the microbial community. Low concentrations may not significantly alter the selective landscape, allowing a broader range of microorganisms to thrive, while higher concentrations may create a more stringent selective environment, favoring only a few highly resistant or adapted microbial species. Finally, nanodrugs can interact with soil components such as organic matter, clay minerals, and nutrients. Low concentrations may enhance these interactions in a beneficial way, improving soil fertility and microbial habitat quality, whereas high concentrations could lead to adverse chemical reactions, reducing nutrient availability or altering soil pH, which negatively impacts microbial communities.

**Table 1:** Assembly and gene predictions.

Sample number	Total contigs	Conting largest contig/bp	Contings total length/bp	N50/bp	N75/bp	GC (%)
CK1	656,690	156,931	275,475,581	889	625	61.82
CK2	555,032	302,373	254,147,055	902	633	62.82
CK3	627,817	336,565	321,457,614	928	640	62.82
A1	833,035	183,526	209,931,876	769	593	60.59
A2	460,833	63,114	180,603,975	817	606	62.8
A3	492,963	76,345	195,150,211	832	610	62.5
B1	477,820	73,118	244,406,647	816	606	61.21
B2	477,551	78,139	195,761,009	880	623	62.87
B3	515,899	63,387	194,823,596	871	621	62.86
C1	474,268	165,545	335,005,088	853	621	61.35
C2	605,423	109,767	202,056,659	808	606	62.38
C3	751,702	97,954	185,439,405	802	605	62.53

Furthermore, we used a Venn diagram to count the number of Unigenes that are shared and unique among multiple sample groups. Supplementary Figure S2 intuitively presents the similarity and specificity in the number of Unigenes between the CK and A groups. This further illustrates that the A treatment has a significant regulatory/promoting effect on the rhizosphere soil of tobacco. Moreover, the CK and A treatment groups share 2,961,498 common genes.

The protein sequences of Unigenes were compared with the NR_meta database using the DIAMOND software (blastp, evalue ≤ 1e^−5^), and the best alignment result for each Unigene was selected for species classification. The taxonomic information at the phylum level was obtained by integrating with the NCBI taxonomic system. We combined the taxonomic information with the abundance information of Unigenes to obtain the abundance of different phyla in various samples.

Subsequently, metagenomic raw sequences were obtained through sequencing on the Illumina platform and then subjected to quality control and assembly. The results are shown in Table 1. A total of 4 soil samples were collected from the rhizosphere of tobacco plants treated with nanodrugs and water (the water treatment and three different drug concentration treatment groups), with the experiment repeated 3 times. After sequence assembly, a total of 6,929,033 contigs were obtained, with each sample having more than 460,000 contigs. The N50 values were all greater than 750 bp, with the maximum reaching 928 bp, and the N75 values were all greater than 600 bp, indicating good assembly quality and suitability for subsequent analysis. The GC content of the total contigs was measured, with an average GC% of 61%, which is within the normal range for gene-level analysis.

### 3.2 Species diversity analysis

#### 3.2.1 Alpha diversity analysis

The Shannon and Simpson indices, which are used to estimate the diversity and species richness of microbial communities, were calculated. The Alpha diversity analysis of soil microbial communities (Table 2) revealed that after the application of nanodrugs via root irrigation, both indices increased, indicating an enhancement in the diversity and species richness of the microbial communities in the treated soil. The Good’s coverage value was 1 for all four samples, suggesting that the sequencing depth was adequate and the species within the samples were comprehensively covered.

**Table 2:** Alpha diversity of soil.

Treatment	Observed species	Shannon	Simpson	Chao1	Goods coverage
CK	29,187 ± 94.53571ab	7.6133 ± 0.01528b	0.95 ± 0b	30184.2533 ± 117.38436ab	1.00a
A	29,145 ± 333.37516ab	7.7967 ± 0.21962ab	0.9533 ± 0.00577b	30147.4433 ± 310.77456ab	1.00a
B	28946.6667 ± 469.8684b	7.7033 ± 0.14434ab	0.95 ± 0b	29960.3833 ± 310.77456b	1.00a
C	29725.3333 ± 396.43074a	7.95 ± 0.14731a	0.96 ± 0a	30675.04 ± 425.859a	1.00a

Different lowercase letters denote significant differences between treatments (*p* < 0.05).

#### 3.2.2 Beta diversity analysis

**Figure 1:**
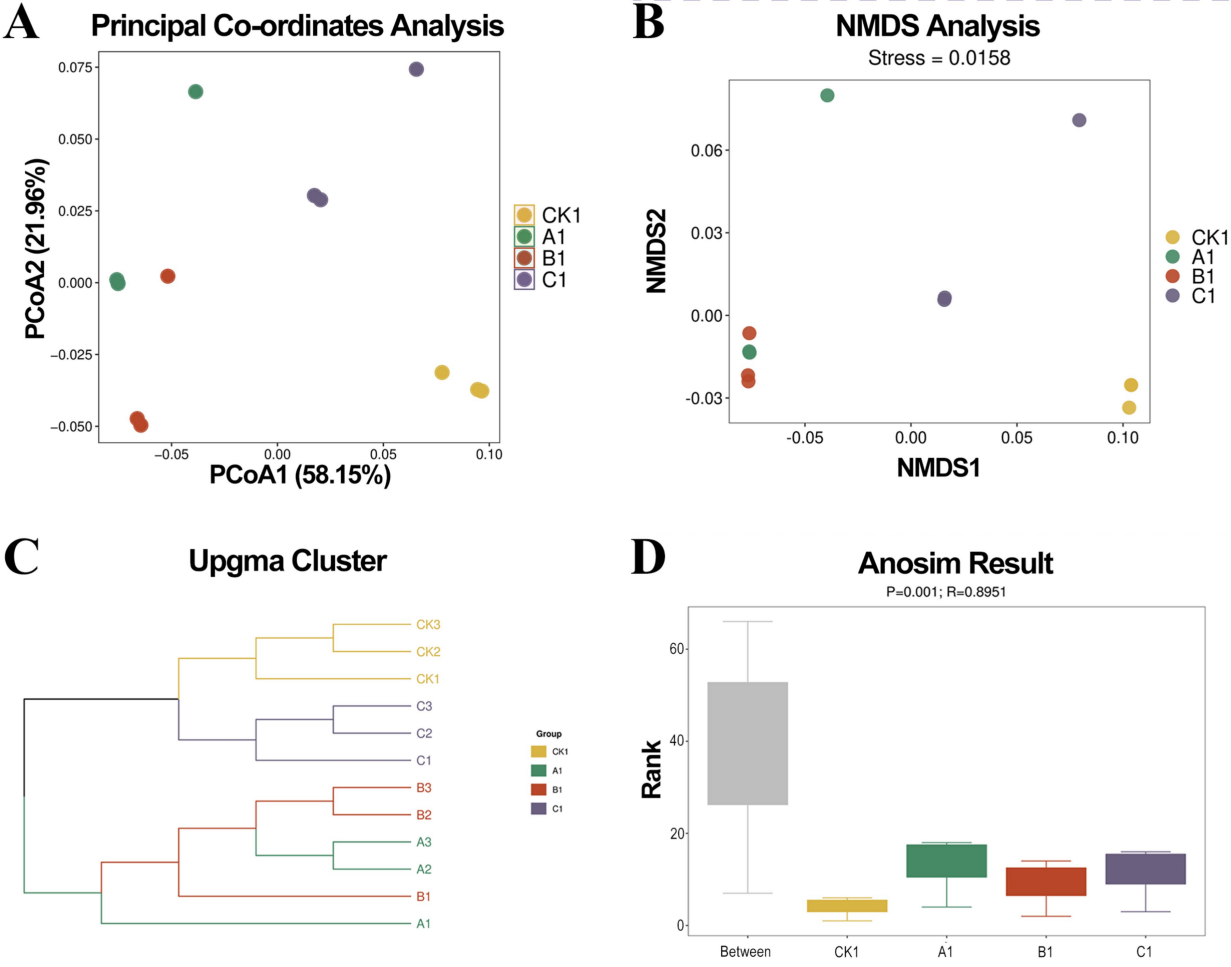
Beta-diversity analysis. **(A)** Principal co-ordinates analysis (PCoA) of soil microbial community structure under different treatments; **(B)** Non-metric multidimensional scaling (NMDS) ordination; **(C)** UPGMA clustering analysis; **(D)** Analysis of similarities (ANOSIM) results.

To investigate the differences in the *β*-diversity of soil bacterial communities, PCoA and NMDS analyses were employed. For an intuitive analysis of the bacterial community structure after the application of nanodrugs, PCoA analysis based on Bray-Curtis distance was conducted (Figure 1A). PCo1 accounted for 58.15% of the community variation, while PCo2 explained 21.96%. The CK group and the A, B, and C treatment groups were located in different quadrants, indicating significant differences in the composition of bacterial communities among the soil samples. The NMDS results (Figure 1B) showed a stress value of 0.0158 for the soil bacterial communities, which is less than 0.05, suggesting that the analysis results are representative. UPGMA (Unweighted Pair Group Method with Arithmetic Mean) clustering was used to construct a hierarchical clustering tree of the samples (Figure 1C). The hierarchical clustering analysis based on NR species composition revealed distinct differences in the composition of microbial communities in soils treated with different concentrations of nanodrugs. The CK group (untreated) and the group treated with 200 μg/mL nanodrugs clustered together, while the A and B treatment groups formed separate clusters with some overlap. This indicates that the soil microbial communities in the CK and 200 μg/mL nanodrug-treated samples were similar, whereas they were distinctly different from those in the A and B treatment groups. The ANOSIM statistical analysis (Figure 1D) showed an R^2^ value of 0.8951, indicating that the differences between groups were greater than those within groups, suggesting a significant impact of nanodrugs on the structure of bacterial communities. The *p* value of 0.001 indicates that there were significant differences in the bacterial communities among the soil samples treated with the three different concentrations of nanodrugs.

### 3.3 Species annotation

#### 3.3.1 Soil microbial abundance

Using the DIAMOND software, a total of 52,375,954 sequences belonging to microorganisms were extracted from the NR database. The NCBI identification included Archaea, Bacteria, Fungi, Virus, and unknown species. As shown in Table 3, bacteria had the highest relative abundance in all soil samples, with a maximum proportion of 86.77% and an average relative abundance of 86.26%. The second most abundant were unknown organisms, with a relative abundance of 10.05–11.13%. The relative abundances of Archaea and Virus were 1.86–3.56% and 0.16–0.42%, respectively, and there were also 0.03–0.07% of eukaryotes. It is evident that bacteria are the predominant microbial group in this soil region, while the contents of Archaea, Fungi, and Virus are relatively low. In addition, all samples contained microbial groups with unclear taxonomic status, with a relative abundance of about 10.00%, indicating a rich biodiversity in the soil region’s ecosystem.

**Table 3:** Statistics on microbial abundance of soils.

Sample number	Bacteria/%	Archaea/%	Eukaryotes/%	Viruses/%	Unknown species/%
CK	86.1367 ± 0.10214a	2.5967 ± 0.11015a	0.0433 ± 0.00577a	0.2167 ± 0.09815a	11.0133 ± 0.10116a
A	86.5067 ± 0.28148a	2.52 ± 0.11a	0.0433 ± 0.00577a	0.2833 ± 0.11846a	10.6467 ± 0.23459ab
B	85.9867 ± 0.14572a	3.26 ± 0.23516a	0.0433 ± 0.02309a	0.19 ± 0.03464a	10.5233 ± 0.04163b
C	86.43 ± 0.58284a	2.9833 ± 0.53326	0.0467 ± 0.01155a	0.2867 ± 0.08963a	10.2533 ± 0.29366b

With regard to the significance labeling of inter-group differences in our study, the letter labeling method was employed for statistical presentation. Specifically, groups marked with the same letter (e.g., a or b) indicate that there was no statistically significant difference in their indicator means at the *p* < 0.05 level; groups labeled with distinct letters represent significant inter-group differences; and groups with combined letters (e.g., ab) suggest no significant differences compared with the groups labeled a or b. This labeling approach is based on the results of multiple comparisons subsequent to analysis of variance (ANOVA), which is a commonly used and standardized method in biological research to intuitively and accurately reflect the significance of differences among treatment groups.

#### 3.3.2 Composition of bacterial communities

**Figure 2:**
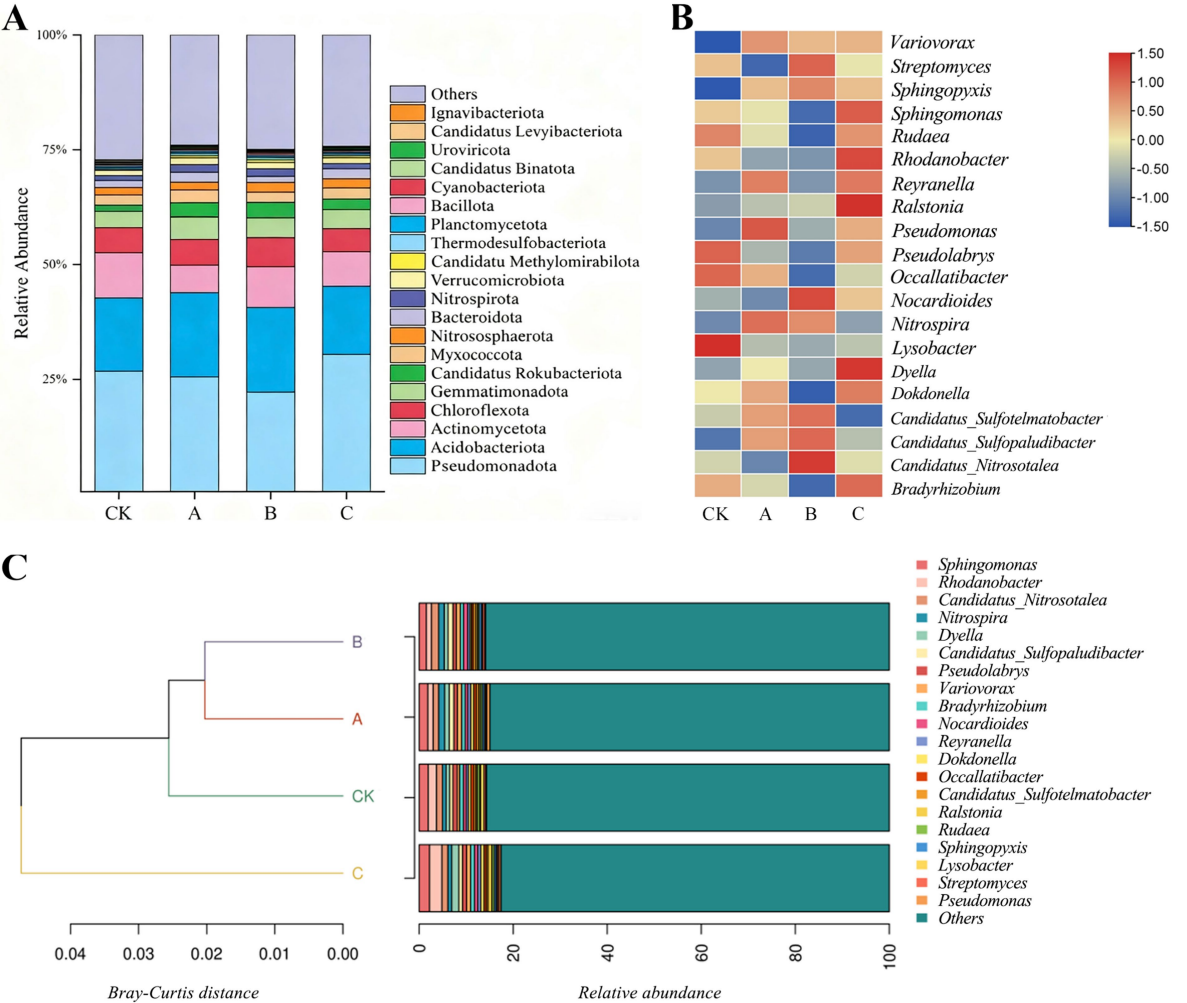
**(A)** Species relative abundance at the phylum level; **(B)** heatmap of soil microbial communities at the genus level across different treatment groups; **(C)** sample clustering analysis.

In the composition of soil microbial communities in the experimental groups A, B, and C, as well as the control group CK, bacteria were the predominant microorganisms. A further analysis was conducted on the bacterial communities. (Figure 2A) illustrates the top 20 most abundant species at the phylum level in each sample. At the phylum level, the dominant bacterial phyla across all samples were Pseudomonadota, Acidobacteria, Actinomycetota, and Chloroflexota, with a combined relative abundance of over 50%. In the CK group, the average relative abundance of Pseudomonadota was 26.76%, Acidobacteria was 15.96%, Actinomycetota was 9.80%, and Chloroflexota was only 5.50%. In the experimental groups A, B, and C, the average relative abundance of Pseudomonadota was 26.25%, Acidobacteria was 17.23%, Actinomycetota was 7.46%, and Chloroflexota was 5.71%. It is evident that the relative abundance of Acidobacteria and Chloroflexota in the soil treated with nanodrugs was significantly higher compared to the control, while the relative abundance of Pseudomonadota and Actinomycetota decreased notably. To further analyze the impact of different nanodrug treatments on the composition of tobacco soil microbial communities, a heatmap was generated to display the top 20 most abundant genera at the genus level (Figure 2B). The results showed certain differences in the relative abundance of species at the genus level among the treatments. Specifically, in the 200 μg/mL treatment, the relative abundance of *Sphingomonas*, *Rhodobacter*, *Dechloromonas*, *Bradyrhizobium*, *Rhizobium*, and *Ralstonia* was higher than in other treatments. In the 150 μg/mL treatment, the relative abundance of *Nitrosomonas*, *Thiobacillus*, *Nocardioides*, *Thiobacter*, and *Streptomyces* was higher than in other treatments. In the 100 μg/mL nanodrug treatment, the relative abundance of *Nitrospira* and *Pseudomonas* was higher than in other treatments. Overall, among the top 20 most abundant genera, most species had a higher relative abundance in the 200 μg/mL nanodrug treatment compared to the 150 μg/mL nanodrug, 100 μg/mL nanodrug, and water treatments.

To investigate the similarities between tobacco plants treated with different concentrations of NMs, we conducted a clustering analysis among samples based on the Bray-Curtis distance matrix. The clustering results were then integrated with the species abundance of each sample and presented together (Figure 2C).

The clustering tree of microbial communities based on Bray-Curtis distance, combined with the stacked bar chart of microbial community composition, illustrates the similarities and differences in community structure among the four samples A, B, CK, and C. As shown in the clustering tree (Figure 2C), B and A first cluster together, followed by clustering with CK, while C forms a separate branch. This indicates that the community structures of B and A are more similar to each other, whereas the community structure of C is significantly different from the other three samples. The stacked bar chart further displays the relative abundance of tobacco microbial genera within different samples.

**Figure 3:**
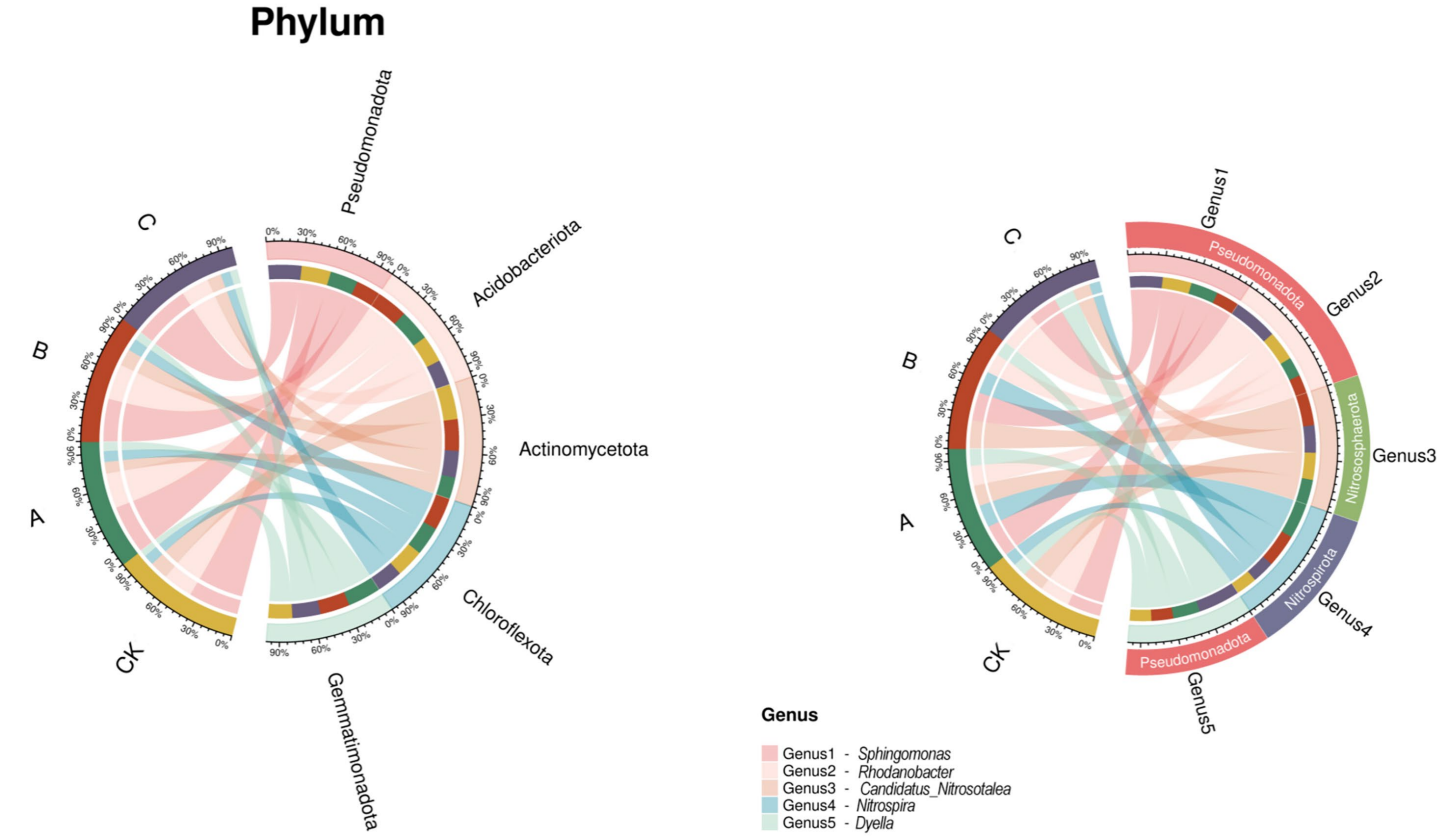
Circos plot showing the top five most abundant species at the phylum and genus levels.

Figure 3 illustrates the relationship between different nanodrug concentrations and the phylum level of tobacco, reflecting the proportion of dominant species composition at different concentrations and the distribution proportion of each dominant species among various concentrations. The top five most abundant species at the phylum level are Pseudomonadota, Acidobacteriota, Actinomycetota, Chloroflexota, and Gemmatimonadota, indicating their status as dominant species. At the genus level, *Sphingomonas*, *Rhodanobacter*, *Candidatus-Nitrosotalea*, *Nitrospira*, and *Dyella* are the top five most abundant species.

**Figure 4:**
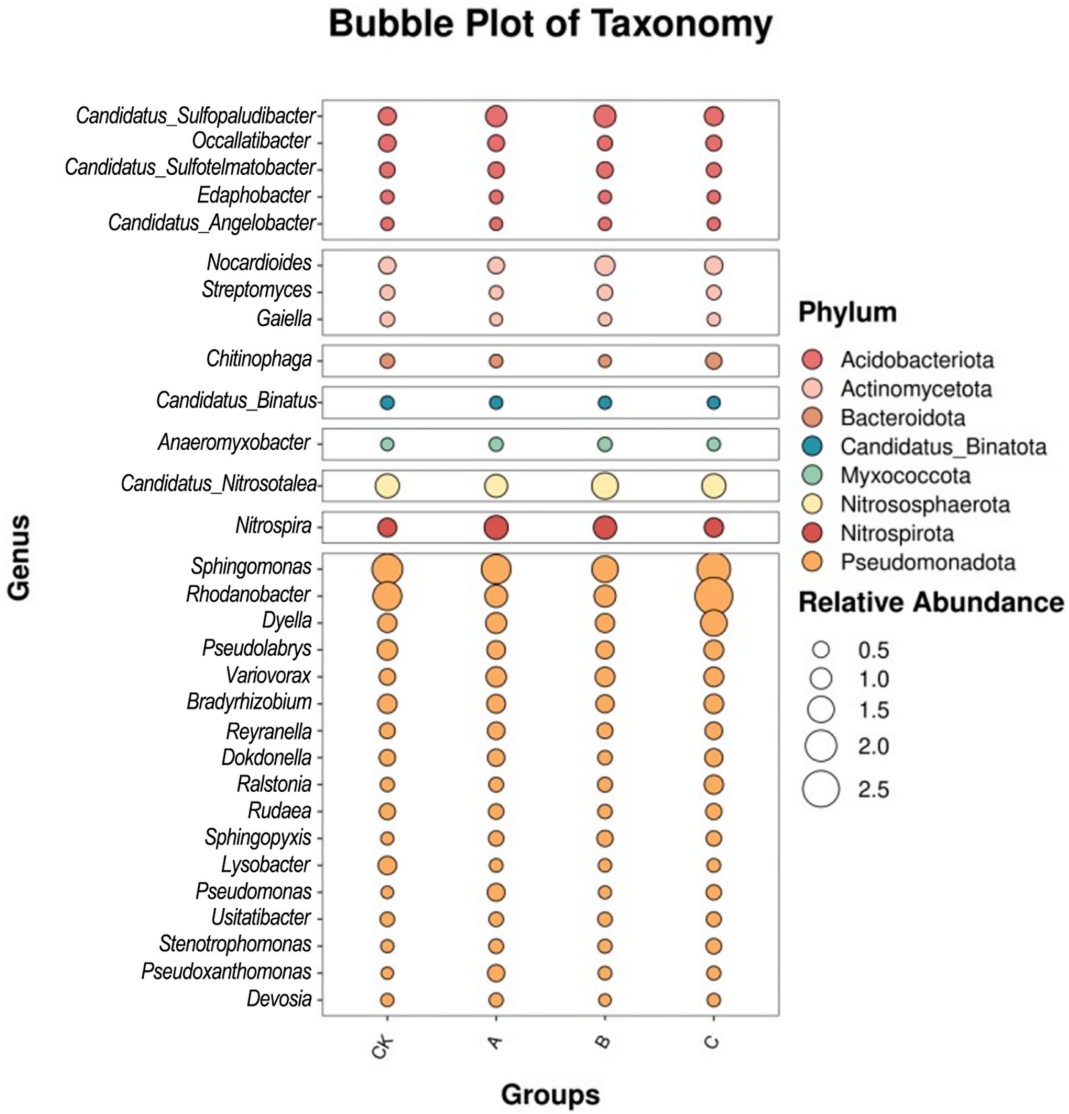
Species bubble chart.

Among the top 30 most abundant genera (Figure 4), the relative abundance of *Sphingomonas* decreased under the B nanodrug concentration, while that of *Candidatus-Nitrosotalea* increased. The abundance of *Rhodanobacter* significantly increased under the C nanodrug concentration. Additionally, the relative abundance of *Nitrospira* decreased with increasing nanodrug concentration. These results indicate that the B concentration inhibits *Sphingomonas* but promotes *Candidatus-Nitrosotalea*. *Rhodanobacter* is more affected by high nanodrug concentrations and shows a positive correlation. *Nitrospira* is negatively correlated with low nanodrug concentrations.

**Figure 5:**
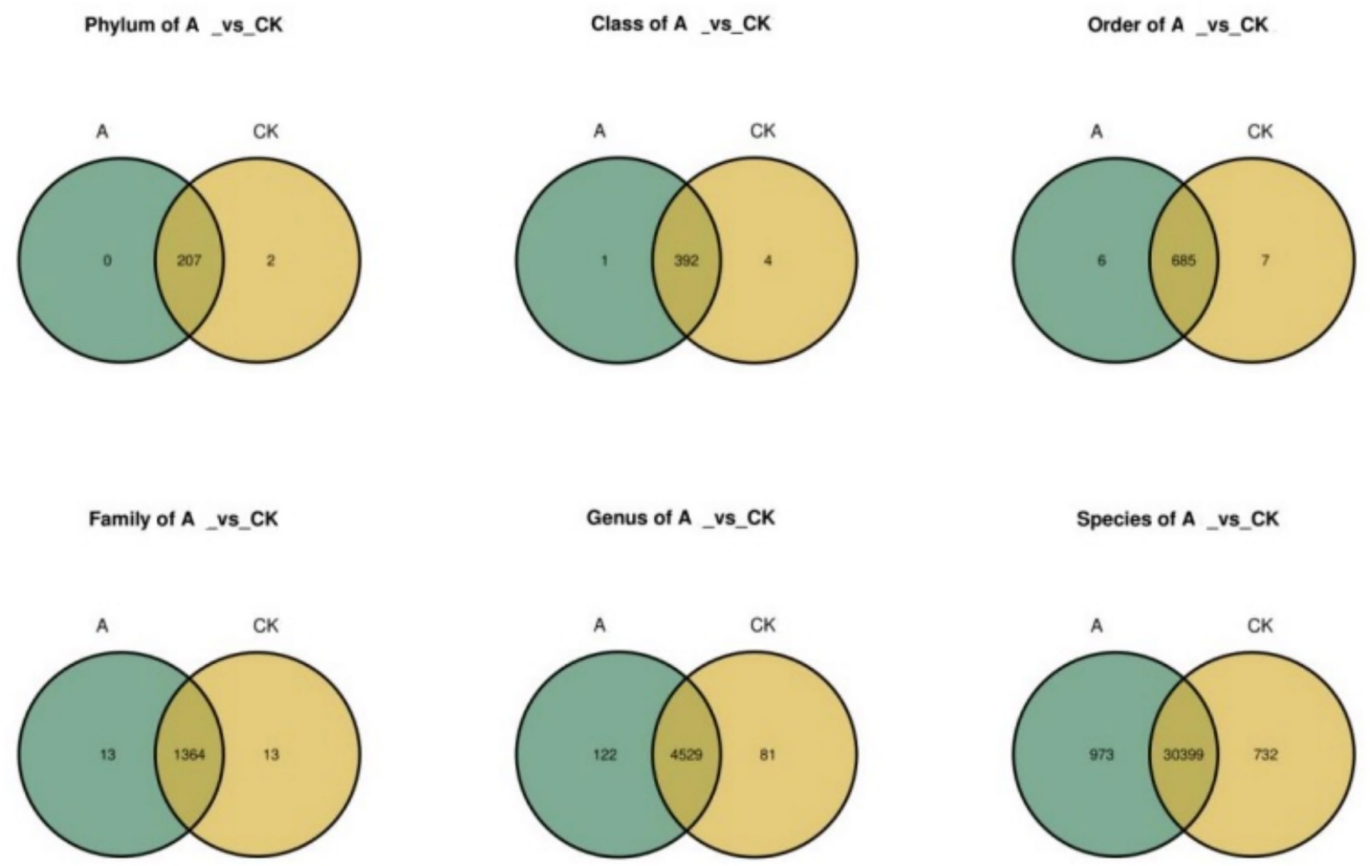
Venn diagram of species composition between the A concentration and the CK control group.

Species Venn diagrams can intuitively display the overlap and uniqueness of species composition among multiple sample groups. Figure 5 shows the species composition at each level between the A concentration and the CK control. At the phylum level, the A and CK control groups share 207 species. Additionally, A has no unique species, while CK has two unique species. At the genus level, A and CK share 4,529 species, with the A treatment group having 122 unique species and the CK control group having 81 unique species. This indicates that the species composition of the A concentration nanomaterial treatment group is more similar to that of CK.

## 4 Analysis of soil microbial community functions

### 4.1 Analysis of gene ontology abundance composition

**Figure 6:**
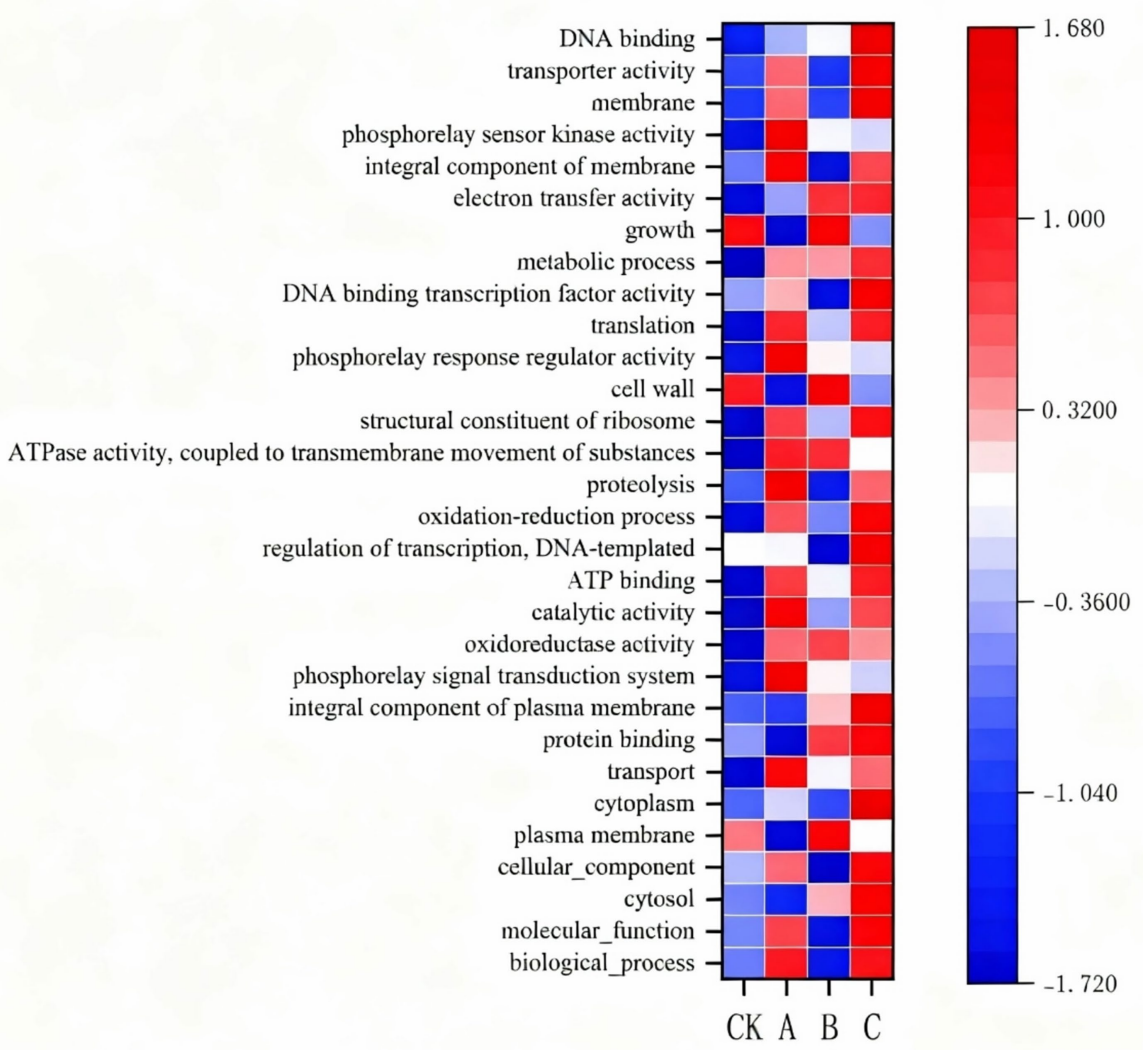
Heatmap of GO enrichment analysis results.

The Gene Ontology (GO) database was employed to predict the functions of the microbial community genes in the rhizosphere soil, aiming to explore the differences in functional distribution between the soil microbial communities treated with nanodrugs and those untreated. The results of functional prediction indicated significant differences between the two (Figure 6). It was found that the main functions enriched in the soil microbial communities treated with nanodrugs were DNA binding, electron transfer activity, metabolic processes, translation, regulation of phosphate relay sensor activity, ribosomal large subunit component, ATPase activity coupled to transmembrane movement of substances, redox processes, ATP binding, catalytic activity, and oxidoreductase activity. In contrast, the main functions enriched in the untreated soil microbial communities were growth, cell wall, cell membrane, and DNA transcription template regulation. Notably, the relative abundance of electron transfer activity and metabolic processes increased with the increasing drug concentration, showing a positive correlation between the strength of these functions in soil microorganisms and the drug concentration.

### 4.2 KEGG functional annotation of soil microbes

**Figure 7:**
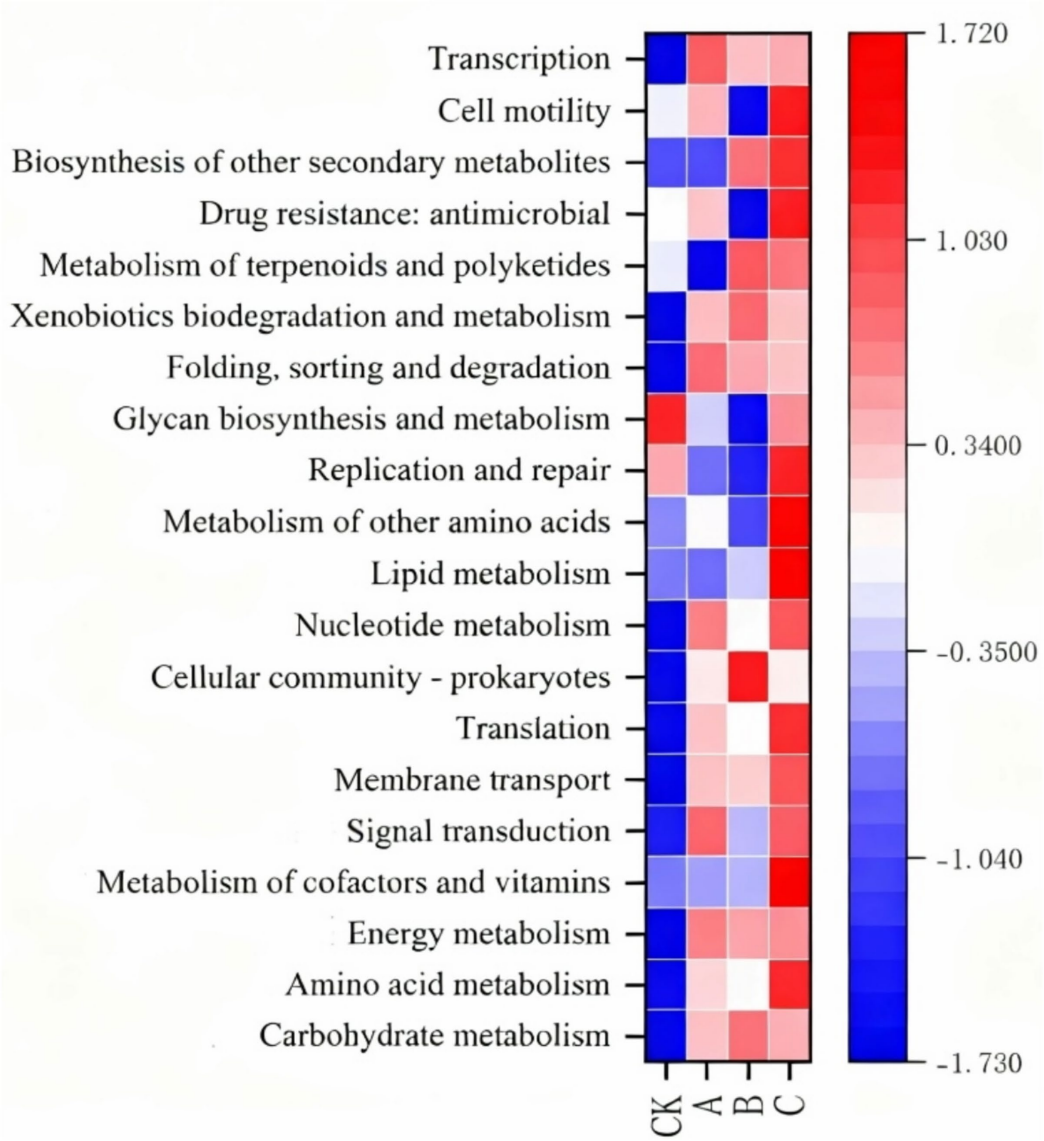
KEGG pathway analysis at level 2.

To investigate the functional distribution differences in soil microbial communities after the application of nanodrugs, the KEGG database was used to perform differential functional analysis on soil microbial genes. The KEGG level 2 results showed that the main functions enriched in the microbial communities treated with nanodrugs via root irrigation (A, B, C) were: transcription, biodegradation and metabolism of xenobiotics, folding, sorting and degradation, nucleotide metabolism, cellular communities-prokaryotes, translation, membrane transport, signal transduction, energy metabolism, amino acid metabolism, and carbohydrate metabolism. In contrast, the functions enriched in the microbial communities in the CK group were: biosynthesis and metabolism of glycan, replication and repair, cell motility, drug resistance: antibacterial, and metabolism of terpenoids and polyketides, as shown in Figure 3. Other pathways such as the biosynthesis of secondary metabolites, membrane transport, and metabolism of cofactors and vitamins showed an upward trend. Since KEGG level 2 is relatively macroscopic and broad, further analysis was conducted using Pathway Definition (Figure 7). The results showed the top 20 most abundant functional pathways annotated in all samples. Among them, the relative abundance of fatty acid metabolism, aminoacyl-tRNA biosynthesis, ribosome, purine metabolism, biosynthesis of cofactors, and metabolic pathways in the tobacco rhizosphere soil microbial communities treated with 200 μg/mL nanodrugs was significantly higher than that in the untreated soil. Notably, fatty acid metabolism and amino acid biosynthesis showed a positive correlation with nanodrug concentration.

**Figure 8:**
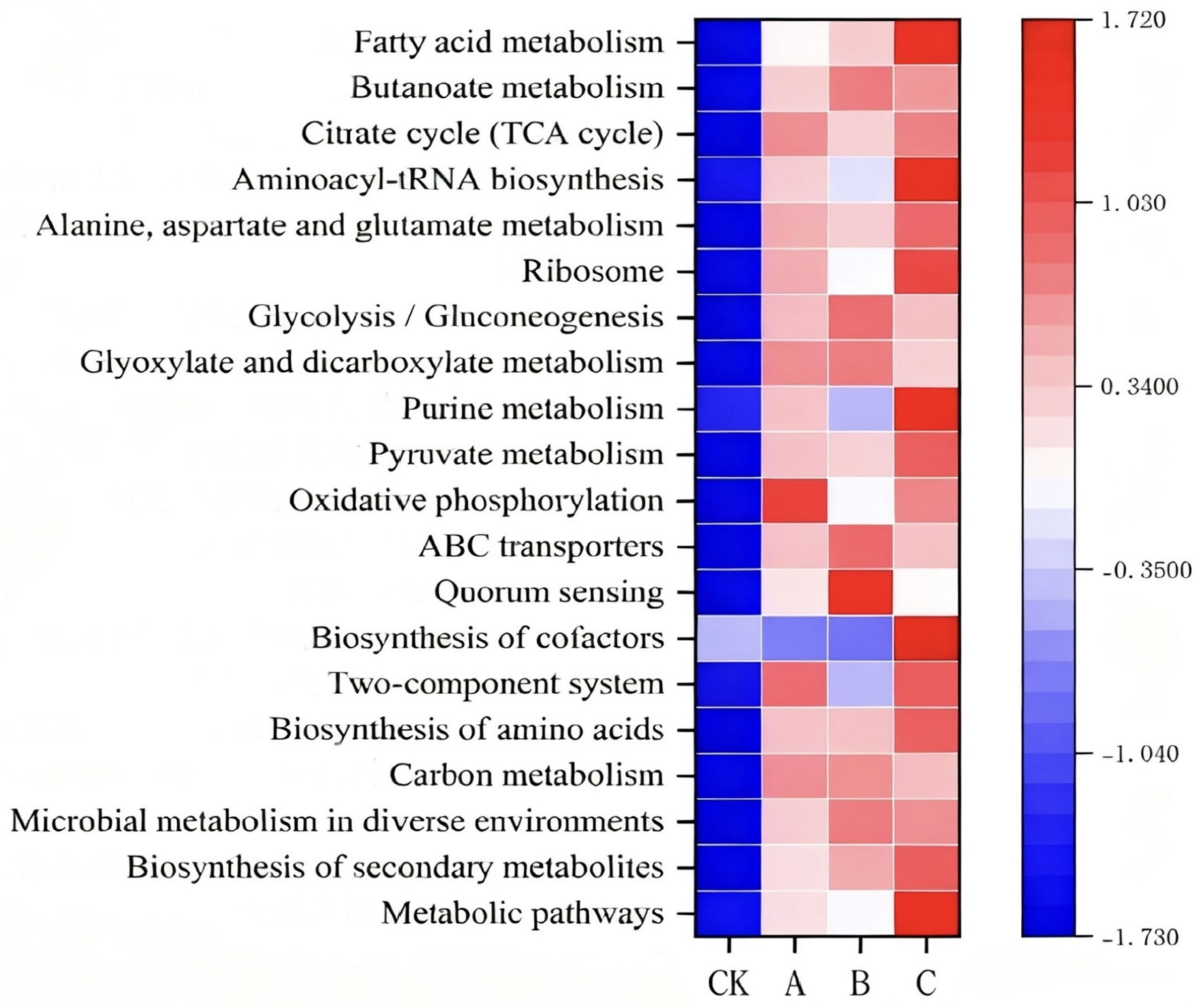
Pathway definition.

To investigate the differences in the functional distribution of soil microbial communities after the application of nanodrugs, differential functional analysis of soil microbial genes was conducted using the KEGG database. The KEGG level 2 results indicated that the main functions enriched in the microbial communities treated with nanodrugs via root irrigation (A, B, C) were transcription, biodegradation and metabolism of xenobiotics, folding, sorting and degradation, nucleotide metabolism, cellular communities-prokaryotes, translation, membrane transport, signal transduction, energy metabolism, amino acid metabolism, and carbohydrate metabolism. In contrast, the functions enriched in the microbial communities in the CK group were glycan biosynthesis and metabolism, replication and repair, cell motility, drug resistance: antibacterial, and metabolism of terpenoids and polyketides, as shown in Figure 3. Other pathways, such as the biosynthesis of secondary metabolites, membrane transport, and metabolism of cofactors and vitamins, showed an upward trend. Since KEGG level 2 is relatively macroscopic and broad, further analysis was performed using Pathway Definition (Figure 8). The results showed the top 20 most abundant functional pathways annotated in all samples. Among them, the relative abundance of fatty acid metabolism, aminoacyl-tRNA biosynthesis, ribosome, purine metabolism, biosynthesis of cofactors, and metabolic pathways in the tobacco rhizosphere soil microbial communities treated with 200 μg/mL nanodrugs was significantly higher than that in the untreated soil. Notably, fatty acid metabolism and amino acid biosynthesis showed a positive correlation with nanodrug concentration.

## 5 Discussion

The rhizosphere microbial community plays a significant role in plant stress resistance and pathogen suppression, acting as a major driver of plant defense responses (Philippot et al., 2013). In this study, metagenomic sequencing revealed that the application of nanodrugs increased the Shannon and Simpson indices in the soil. Similar findings have been reported in studies where nano-selenium treatment also raised the bacterial Chao1 index (Zhu et al., 2025), which aligns with the results of this research. This may be attributed to the strong adaptability and resistance of certain bacteria. PCoA analysis showed that the CK group and the nanodrug treatment groups were distributed in different quadrants (Figure 1A), which well explains that nanodrugs may play a key role in shaping microbial community structure. This finding is consistent with other studies on NMs (He et al., 2011). The impact of nanodrugs on soil microbial communities may be related to their inherent properties. Due to their small size, stability, and targeted delivery capabilities, NMs can easily be transported into the soil to interact with soil microorganisms (Lowry et al., 2024). NMDS and ANOSIM analyses further validated the PCoA results. This is in line with the findings of Jiao et al. (2017) on the effects of nano-silver on soil microorganisms. The UPGMA clustering results in this study showed that the high-concentration nanodrug treatment (C) clustered with the CK group (Figure 1C), while the medium- and low-concentration treatments (A, B) formed separate clusters with some overlap. This suggests that high concentrations of nanodrugs may inhibit or select for certain microbial groups, leading to a community structure more similar to the control group. In contrast, medium- and low-concentration treatments may trigger more complex community responses, such as adaptive growth or competitive replacement of certain groups. This discovery indicates that the impact of nanodrugs on microbial communities may have a certain concentration effect.

Analysis of the microbial community revealed that Pseudomonadota and Acidobacteria are the dominant groups (Figure 3). Pseudomonadota plays a significant role in global carbon, nitrogen, and sulfur cycles and can promote plant growth by directly inhibiting pathogens, secreting auxins, and producing siderophores (Kersters et al., 2006). Acidobacteria are acid-tolerant groups in the soil and are oligotrophic microorganisms that can enhance crop growth and development by regulating soil physicochemical properties (Liu et al., 2016; Kalam et al., 2022). Further analysis showed that nanodrug treatments increased the relative abundance of *Sphingomonas*, *Rhodobacter*, *Dechloromonas*, *Bradyrhizobium*, *Rhizobium*, *Ralstonia*, *Nitrosomonas*, *Thiobacillus*, *Nocardioides*, *Thiobacter*, *Streptomyces*, *Nitrospira*, and *Pseudomonas*. These microorganisms all have unique functional potentials. *Sphingomonas* is known for its ability to degrade hydrocarbons and is closely related to the utilization of plant root exudates (Festa et al., 2016). It has shown significant control effects on bacterial wilt in tomatoes and sheath blight in rice (Wang et al., 2025; Matsumoto et al., 2021). *Bradyrhizobium* is an important free-living diazotroph with nitrogen transformation and soil organic carbon accumulation functions (Tao et al., 2021). *Pseudomonas* can produce siderophores to inhibit disease occurrence (Shao et al., 2024). *Nocardioides* bacteria play a significant role in biological nitrogen fixation, phosphate solubilization, and siderophore production (Nouioui et al., 2022). Some studies have found that nano-selenium (SeNPs) can specifically enrich beneficial rhizosphere bacterial communities, such as increasing the abundance of Bacillus in rice or inhibiting fungal infections in rapeseed (Jiao et al., 2023; Han et al., 2024). Carbon nanosols (CNS) significantly alter rhizosphere microbial composition by enriching beneficial bacteria like *Sphingomonas* and *Burkholderia* to promote tobacco growth (Cheng et al., 2024). Therefore, nanodrugs may create a microenvironment unfavorable for pathogen growth and selectively promote the proliferation of beneficial microorganisms, thereby establishing a healthier rhizosphere microecosystem.

Through functional prediction analysis using the GO database in this study, it was observed that functions related to energy metabolism and electron transfer (such as electron transfer activity, ATPase activity, and redox processes) were significantly enriched in the nanodrug treatment groups. Electron transfer activity is central to microbial energy synthesis, being especially closely related to the respiratory chain and ATP synthesis (Hao et al., 2025). The enhancement of metabolic processes, including the metabolism of carbohydrates, amino acids, and fatty acids, provides microorganisms with ample energy and substrates, supporting their growth and stress responses (Chen et al., 2022a). The activation of these functions may be related to the special surfaces or catalytic properties provided by nanodrugs. For example, graphene-based NMs have been proven to mediate electron transfer and enhance the efficiency of microbial redox reactions (Du et al., 2020). Moreover, the enrichment of ATPase activity and ATP binding indicates enhanced capabilities of microorganisms for transmembrane transport and energy utilization, which may aid in nutrient absorption and toxin efflux, thereby improving microbial stress tolerance (Xiao et al., 2025). Additionally, this study found that the functional abundance of electron transfer activity and metabolic processes was positively correlated with nanodrug concentration. This suggests that the effects of nanodrugs are dose-dependent. Previous studies have shown that carbon NMs can promote soil microbial metabolism at low concentrations, while high concentrations may inhibit certain microbial groups but enhance overall functional redundancy (Ahmed et al., 2023). This dose-dependent effect may be related to the surface area and reactivity of nanoparticles: higher concentrations provide more interfaces for microbe-nanoparticle interactions, thereby enhancing electron transfer and catalytic reactions (Xiao et al., 2025). These findings suggest that nanodrugs may enhance their ecological functions and indirectly promote plant health by regulating the core metabolism and energy production pathways of soil microorganisms.

The KEGG functional annotation analysis of soil microorganisms revealed that the relative abundance of several key metabolic pathways in the tobacco rhizosphere soil of the nanodrug treatment groups was significantly increased. These pathways included fatty acid metabolism, aminoacyl-tRNA biosynthesis, ribosome, purine metabolism, cofactor biosynthesis, and basic metabolic pathways. The enhancement of fatty acid metabolism is likely closely related to the synthesis of antimicrobial substances, as many antimicrobial compounds are biosynthesized from fatty acid precursors. Similarly, the strengthening of amino acid biosynthesis provides sufficient substrates for the production of secondary metabolites, which include antimicrobial substances. Fatty acids not only constitute the main components of microbial cell membranes but also participate in signal transduction and energy storage processes. Studies have shown that the composition of fatty acids in tobacco rhizosphere soil microorganisms is closely related to microbial community structure, with specific increases in fatty acids significantly correlated with bacterial growth promotion (Gong et al., 2024). Silver nanoparticles can alter soil sugar and amino acid-related metabolic pathways, thereby affecting carbon and nitrogen metabolism processes (Zhang et al., 2020). Therefore, nanodrugs may regulate the metabolism of these key fatty acids, strengthen the structure of microbial cell membranes, enhance microbial resistance to environmental stress, and thus maintain the stability of the rhizosphere microbial community. On the other hand, the activation of aminoacyl-tRNA biosynthesis and ribosome pathways indicates that nanodrug treatment promotes the protein synthesis mechanism of microorganisms. In studies on the impact of water and nitrogen interactions on tobacco metabolism, it was found that the enhancement of the aminoacyl-tRNA biosynthesis pathway is closely related to plant stress resistance. This pathway not only affects protein synthesis efficiency but also participates in cellular stress responses and signal transduction processes (Ostersetzer-Biran and Klipcan, 2020). This finding corroborates our results, suggesting that nanodrugs may enhance microbial metabolic activity and adaptability by strengthening their protein synthesis capabilities. The activation of purine metabolism and cofactor biosynthesis provides energy and cofactor support. Purines are precursors for the synthesis of genetic material, while cofactors (such as vitamins and coenzymes) are key to many catalytic reactions. The enrichment of these pathways may maintain a high metabolic activity state in microorganisms. A study found that purine metabolism is significantly enriched in tobacco stress responses, with significant differences in the content of metabolites in the nicotine synthesis pathway (Xia et al., 2025). These enhanced metabolic functions together constitute a “metabolic barrier” for microorganisms to inhibit pathogen invasion, effectively reducing the risk of tobacco diseases through nutrient competition, spatial competition, and the production of specific antimicrobial substances. This tripartite interaction mechanism of “NMs-microbes-plants” paves a new way for microbiome engineering.

## 6 Conclusion

This study describes a novel strategy for alleviating tobacco diseases using nanodrugs. Our research indicates that nanodrugs can modulate the structure of soil microbial communities and selectively recruit microorganisms with potential functional capabilities. Moreover, nanodrugs may also activate key metabolic pathways such as fatty acid metabolism and purine metabolism in these indigenous microorganisms to assist tobacco in disease control. These results suggest that the application of nanodrugs effectively optimizes the rhizosphere microecological balance, enhances biocontrol efficacy, and activates plant endophytic defense, providing a micro-level pathway for disease control via the synergy between NMs and microorganisms. This study provides a foundation for further research on the use of nanopesticides as a strategy to promote plant health in sustainable agricultural systems.

## Data Availability

The original contributions presented in the study are included in the article/Supplementary material, further inquiries can be directed to the corresponding author/s.
