# Supplementary material for: The impact of nanodrugs on the metagenome of tobacco rhizosphere soil
Source: Front Microbiol. 2026 Feb 11;16:1715400. doi: 10.3389/fmicb.2025.1715400 (PMC12932420; doi:10.3389/fmicb.2025.1715400)
Supplement: Supplementary file 1 [file Image_1.pdf]

The Impact of Nanodrugs on the Metagenome of Tobacco Rhizosphere Soil

Chun-Mei Lai[1, #], Xiao-Shan Xiao[1, #], Li-Wei Liu[1, #], Xue-Li Li[1], Yu-Wei Luo[1], Yan-Qi Liang[1], Yan Cheng[1, *], Yuan Qin[1, *]

[1]College of Life Sciences, College of Plant Protection, Fujian Provincial Key Laboratory of Haixia Applied Plant Systems Biology, Fujian Agriculture and Forestry University, Fuzhou, 350002, China.

***Corresponding Author**: Yan Cheng and Yuan Qin

Email: chengyan1220@hotmail.com; Yuanqin@fafu.edu.cn

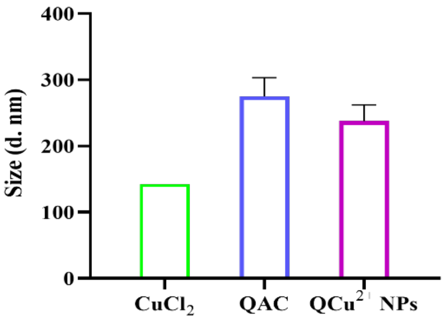

Fig. S1. The particle size of QCu^{2+} NPs and the corresponding counterparts.

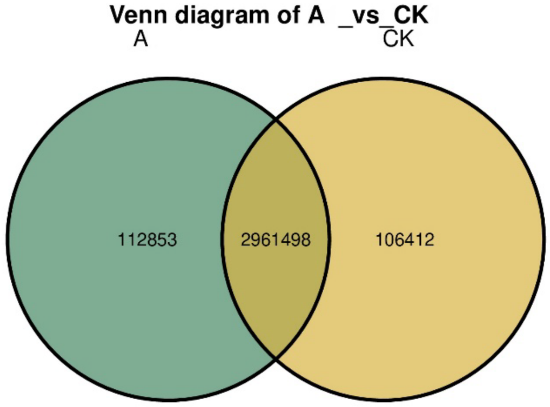

Fig. S2. Venn diagram of the number of single genes.
